# IgG4-related disease and prostate cancer: A case report and review of the literature

**DOI:** 10.1016/j.eucr.2023.102538

**Published:** 2023-08-19

**Authors:** Adam Zekhnini, Daniel Taussky, Jean-Baptiste Lattouf, Martial Koenig

**Affiliations:** a Department of Radiation Oncology, Centre hospitalier de l’Université de Montréal, Montréal, Canada; b Division of Urology, Department of Surgery, Centre Hospitalier de l’Université de Montréal (CHUM), Montréal, Canada; c Internal Medicine Service, Centre hospitalier Universitaire de Montréal (CHUM), Montréal, Québec, Canada

**Keywords:** IgG4, Prostate cancer, Rituximab

## Abstract

We present a case of a patient with recurrent prostate cancer after treatment for favorable intermediate risk cancer. There was an exceptionally steep increase in PSA from <0.5 to 130ng/mL in 27 months accompanied with the development of bone metastasis. The PSA increase was unexpected. We suspect that this unusual development of metastases must have been caused by an impairment of the immune system caused by his IgG4 disease, and this may have allowed residual prostate cancer cells in the prostate to spread quickly. The influence of IgG4 on cancer is debated.

## 1 Introduction

The possibility of an increased malignancy risk in IgG4-RD patients remains controversial. IgG4-related disease (IgG4-RD) is a rare systemic immune-mediated fibro-inflammatory disorder, defined pathologically by a triad of lymphoplasmacytic and IgG4 plasma cell infiltration, storiform fibrosis, and obliterative phlebitis.^1^ The pathogenesis of IgG4-RD remains incompletely understood with no specific autoantigen identified, but growing evidence suggests that CD4^+^ cytotoxic lymphocytes orchestrate apoptosis in the affected tissues. IgG4-RD develops as focal masses and/or diffuse enlargement of target organs, with common phenotypes including autoimmune pancreatitis (AIP), retroperitoneal fibrosis/aortitis and sclerosing sialadenitis/dacryoadenitis and lymphadenopathy.^1^ These formerly thought to be independent clinical entities have since been recognized as clinical manifestations of the same systemic autoimmune condition.

## 2 Case report

**Fig. 1:**
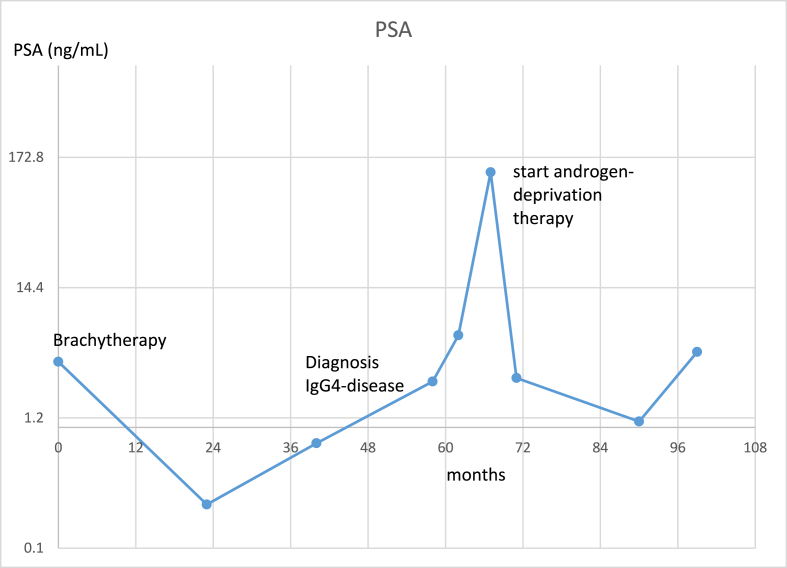
PSA before and after brachytherapy and occurrence of IgG4-related disease with influence on PSA. PSA scale is logarithmically transformed.

The patient was treated at age 70 with brachytherapy for favorable intermediate risk prostate (clinical T1c, PSA 3.54 ng/mL, Gleason 3 + 4 disease in 2/12 biopsies). The brachytherapy implant was inserted with a good quality index (D90 = 192 Gy at day 30). PSA levels declined following the usual rate with a nadir of 0.23 at 24 months post brachytherapy. PSA levels were at 0.29 at 3 years post brachytherapy and some months later top 0.47. Around this time and unrelated to this discrete increase in PSA, the patient was hospitalized with hepatobiliary symptoms. See Fig. 1 for a PSA trend over time with time of diagnosis of IgG4 disease and start androgen deprivation therapy. His abdominal CT showed diffuse pancreatic edema, renal lesions and a large abdominal paraaortic tissular infiltration which let to suspect Ig-G4 related disease. At 4 years PSA levels were 0.74, with a sudden increase to 5.8 at 5 years post brachytherapy. Six months later, PSA levels skyrocketed to a high of 130, corresponding to a 277-fold PSA level increase within 27 months, from 0.47 to 130. Approximately two months before PSA levels of 130 were detected, an abdominal CT-scan proved negative for metastatic lymphnodes. Because of the sharp rise in PSA and the concern for metastatic disease development, androgen deprivation therapy (ADT) in the form of Leuprolide was started immediately. On a follow-up CT scan and bone-scan prescribed for IgG4-related symptoms, multiple bone metastases were identified within 4 months of the 130 PSA value and enzalutamide was started. These metastases were most likely already present at the 130 PSA value time, before the start of ADT. Under ADT, a PSA level nadir of 0.18 was reached, with subsequent rise about 12 months post enzalutamide with tripling of PSA within 6 months but associated with radiologic regression of bone metastases.

Concerning his IgG4disease, a scan showed kidney and lung masses, as well as pancreatic masses causing biliary obstruction. A pancreatic biopsy demonstrated fibrosis with lymphoplasmocytic infiltrate and venulitis without thrombosis. The IgG4/IgG positive plasmocyte ratio was normal. IgG4 serum levels were 1,6 g/L (upper limit 1.35 g/L). Therefore, a preliminary diagnosis of IgG4 related-disease was established and prednisone was started at 50 mg p. o. (0,6 mg/kg) leading to a rapid clinical and radiological improvement. IgG4 levels dropped to 0,778 g/L. Following this, prednisone was slowly tapered with recurrence of previously reported abdominal pain and jaundice, as well as radiological progression of the pancreatic masses. A pancreatic biopsy and an elevated IgG4:IgG ratio (>40), together with a IgG4 serum level of 1.92 g/L, confirmed the recurrence of IgG4 related disease (IgG4-RD).

Replacement of glucocorticoids with rituximab or methotrexate was discussed, but Methotrexate was selected for health insurance purposes at 25 mg weekly subcutaneously. Glucocorticoid treatment was slowly tapered to 5 mg. The patient remains relapse free to this day, more than 2 years after initiation of methotrexate treatment.

## 3 Discussion

Some studies suggest an increased risk of malignancies particularly in the first year after IgG4-RD diagnosis.^1^^,^^2^ Most malignancies developed in organs other than those targeted by IgG4-RD inflammation and fibrosis. It is possible that IgG4 antibodies are implicated in carcinogenesis,^3^ suggesting that they contribute to a tumor-associated escape from immune surveillance that stems from IgG4's unique immunomodulatory properties. Such findings, if confirmed, may have clinical applications in cancer immunotherapy regimens. Autoimmune pancreatitis (AIP), a common manifestation of IgG4-RD, has been associated with a significant number of new diagnoses of cancer within a year of diagnosis, about 50% of which are gastric, lung and prostate cancer.

Our case describes rapid increase in PSA after a satisfactory nadir of 0.23 in a favorable intermediate risk cancers. Such increases are very unusual. We speculate that this unusual increase in PSA was due the impaired immune-system caused by his IgG4 disease. We can't prove any such causality and no molecular tests were done to prove such a relationship.

IgG4-RD tends to respond well to glucocorticoids in its active inflammatory stage, Second-line treatments include immune-suppressants such as rituximab, azathioprine and methotrexate.^1^

Rituximab could be an interesting agent to treat both IgG4-RD and his prostate cancer. It is commonly used in the treatment of hematological neoplasms such as CD20-positive leukemia and lymphoma. Rituximab is an anti-CD20 monoclonal antibody and selective B-cell depleting drug. A potential mechanism for such a response involves interference in the maintenance of CD4^+^ T cell memory as a result of rituximab-induced B cell depletion, highlighting the essential role for CD4^+^ cytotoxic lymphocytes in the pathogenesis of IgG4-RD. The role of rituximab in prostate cancer treatment has not yet been established as most antibody immunotherapies have not succeeded in prostate cancer. A 2020 clinical trial, the first such study in eight high-risk prostate cancer patients, neoadjuvant rituximab decreased B-cell and T-cell density in biopsy samples compared to controls, providing evidence that rituximab has the ability to modify the tumor microenvironment in prostate cancer.^4^ Furthermore, a 2018 case report also presents the case of a patient achieving remission of metastatic castrate-resistant prostate cancer following rituximab and bendamustine treatment for a concomitant follicular lymphoma.^5^ These promising, but preliminary findings underline the need to further investigate the potential effect of rituximab in prostate cancer management.

In conclusion, the presented case shows a fulminant evolution of a prostate cancer with a relatively low aggressiveness treated curatively initially. The rapid rise in PSA and rapid development of metastasis could be due an impairment of the immune system. It can't be excluded that there was much more aggressive cancer in the prostate that was missed on initial biopsy because the patient didn't have a diagnostic MRI before treatment.

## Declaration of competing interest

None.
